# Molecular Beacon DNA Probes with Fluorescein Bifluorophore

**DOI:** 10.1134/S1068162021030055

**Published:** 2021-06-11

**Authors:** V. A. Brylev, I. L. Lysenko, E. A. Kokin, Y. V. Martynenko-Makaev, D. Y. Ryazantsev, V. V. Shmanai, V. A. Korshun

**Affiliations:** 1 Shemyakin–Ovchinnikov Institute of Bioorganic Chemistry, 117997 Moscow, Russia; 2 Institute of Physical Organic Chemistry of NAS Belarus, 220072 Minsk, Belarus; 3 Department of Biology and Biotechnology, National Research University Higher School of Economics, 117312 Moscow, Russia; 4 Gause Institute of New Antibiotics, 119021 Moscow, Russia

**Keywords:** fluorogenic DNA probes, 5-carboxyfluorescein, 3,5-diaminobenzoic acid, fluorescence quenching, real-time qPCR

## Abstract

An azido-derivative of a fluorescein bifluorophore was obtained and used for the synthesis of “molecular beacon”-type oligonucleotide fluorogenic probes for RT-PCR. Eight probe variants were synthesized based on an optimized sequence: with one or two quencher residues at the 3'-end, with a single or bifluorophore fluorescein label attached to 5'-end using modifying phosphoramidites (short linker) or “click reaction” (long linker). Comparison of probes in RT-PCR showed that probes with a doubled quencher (single fluorescein on a short linker) and doubled dye on a short linker (single dye) are somewhat superior in sensitivity to a standard probe (single quencher, single dye on a short linker) by the value of ΔC_t_ = 1–2.

**Supplementary Information:**

The online version contains supplementary material available at 10.1134/S1068162021030055.

## INTRODUCTION

Fluorescent DNA probes, which are part of reagent kits for qualitative and quantitative detection of DNA and RNA, continue to be a powerful research and diagnostic tool [1–4]. One of the most important areas of application of such probes is the real-time polymerase chain reaction (RT-PCR) [5]. This method is used for express detection and semiquantitative (to the order of magnitude) analysis of genetic material; the most common uses of RT-PCR are molecular diagnostics of inherited diseases, genetically modified organisms, microbial and viral pathogens, for example, HIV [6] and SARS-CoV-2 [7]. In RT-PCR, various types of fluorogenic DNA probes are used, which are capable of increasing fluorescence when interacting with the accumulating PCR product; the fluorogenic effect is achieved as a result of the interaction of two dyes, one of which can be nonfluorescent (quencher) [5, 8]. For fluorogenic probes, the relationship between the type of dye and the structure of the probe is being studied [9], new dyes are being developed [10–12], and probes with two residues of a fluorescent dye and/or a fluorescence quencher are being investigated [13–15]. The most popular dye for DNA probes is fluorescein, which is attached in the form of a carboxyl derivative at the amino group of a linker; such fluoresceinamide is abbreviated as FAM. Introduction of several fluorescein residues into the bioconjugate can lead to significant self-quenching of fluorescence [16]; on the other hand, attachment of fluoresceins with a rigid linker prevents self-quenching [17]. Earlier, we obtained FAM bifluorophores based on 3,5-diaminobenzoic acid [18, 19]. The purpose of this work was to study FAM-bifluorophore on various linkers compared to a single fluorescein label as part of fluorogenic molecular beacon oligonucleotide probes for RT-PCR.

## RESULTS AND DISCUSSION

**Fig. 1.**
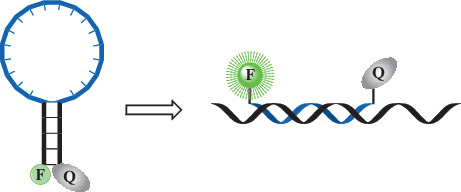
The principle of operation of a fluorogenic molecular beacon DNA probe. F, fluorescent dye; Q, quencher.

DNA probes of the molecular beacon type (Fig. 1) are an oligonucleotide carrying a fluorescent dye (F) and a quencher (Q) at the 5'- and 3'-ends, while the 5'- and 3'-end regions of the probe (5–7 nucleotides) are complementary. As a result, in an aqueous solution at room temperature, the probe exists predominantly in the form of a hairpin structure consisting of a stem and a loop, with the fluorophore and quencher located close to each other. The loop part of the molecular beacon is complementary to the target sequence (in RT-PCR, the PCR product), and, as a result of hybridization with it, the hairpin is destroyed, the dyes are uncoupled, and fluorescence flares up (Fig. 1). Monitoring of the fluorescence intensity is carried out at each PCR cycle, recording as a result the dependence of the emission on the number of PCR cycles.

**Fig. 2.**
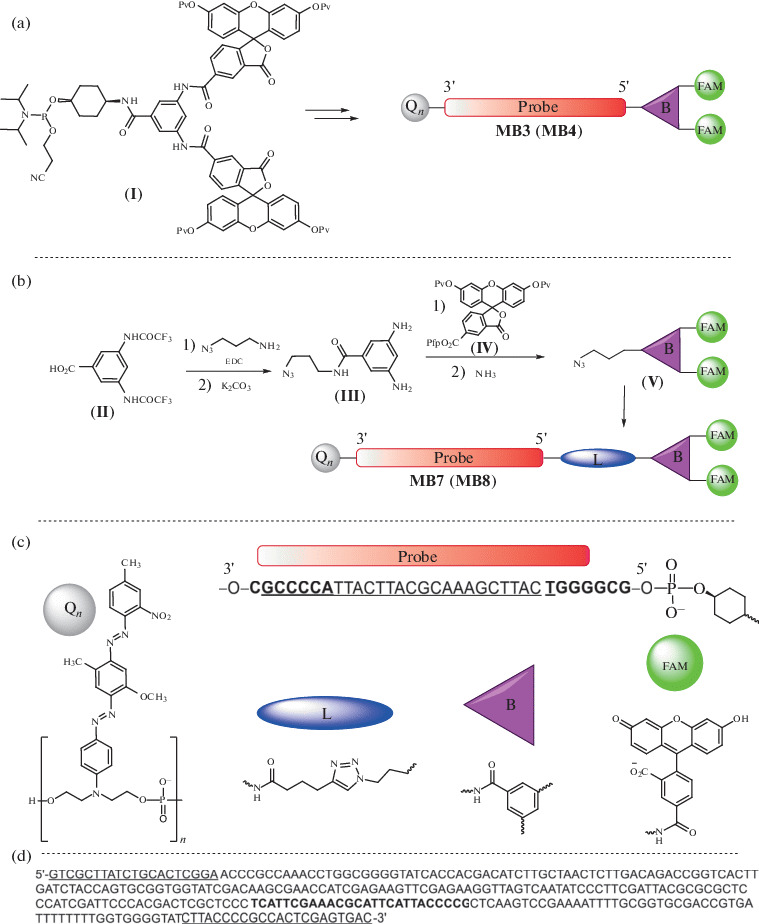
Synthesis of oligonucleotide probes with 5-FAM-bifluorophore. Pv, pivaloyl; Pfp, pentafluorophenyl; FAM, 5-FAM fluorophore. (a) Probe synthesis schemes **MB3** and **MB4** using the amidophosphite method; (b) synthesis of the azido derivative (**V**) and probes **MB7** and **MB8**; (c) the main components of the probe, Q, quencher; L, linker; B, the branching fragment based on 3,5-diaminobenzoic acid. The sequence of the probe complementary to the target is underlined; the fragments that form the stem of the hairpin are highlighted in bold; (d) detected sequence of a 290-bp fragment of the gene encoding translation elongation factor 1α of *Fusarium avenaceum.* Areas complementary to primers Fat65R and Fat65F are underlined; the section of the gene that binds to probes **MB1**–**MB8** is highlighted in bold.

In this work, we used an optimized hairpin probe complementary to the region of the amplified fragment of the fungal translation factor 1α gene *Fusarium avenaceum* [14]. The nucleotide sequence of the probe, the structures of its chemical modifications, and approaches to the synthesis of 5-FAM-bifluorophore probes are shown in Fig. 2. Probes **MB3** and **MB4** (Table 1) were synthesized using phosphoramidite reagent (**I**) [18]. In this work, an azide reagent (**V**) based on 5-FAM-bifluorophore for labeling alkyne-modified oligonucleotides using Cu(I)-catalyzed cycloaddition reaction (click reaction) was obtained. The starting 3,5-bis(trifluoroacetylamino)benzoic acid (**II**) [20] was coupled with 3-azidopropylamine [21], followed by the deprotection of amino groups with ammonia. The resulting diamine (**III**) was acylated with pentafluorophenyl ether of dipivaloyl-protected 5-carboxyfluorescein (**IV**) [22]. Removal of pivaloyl protecting groups led to the formation of an azide derivative of 5-FAM-bifluorophore (**V**). The latter was used to modify alkyne oligonucleotides in solution using a click reaction to afford probes **MB7** and **MB8** (Table 1). Single fluorescein probes were obtained using 5-FAM-phosphamidite [22] (**MB1** and **MB2**) or click modification of alkyne oligonucleotides with a 5-FAM-azide reagent [23] (**MB5** and **MB6**) (Table 1). Terminal alkyne was introduced into oligonucleotides using a phosphamidite reagent [24]. The doubled quencher BHQ1 (Q_2_) was introduced into oligonucleotides as described earlier [13]. All probe components are shown in Fig. 2. One or two BHQ1 (Q) quenchers are located at the 3'-end, while the 5'-end may have one or two fluorophores (5-FAM) attached using phosphamidites or the click-reaction with azide reagents.

**Table 1.** Main characteristics of fluorogenic probes for RT-PCR

Probe structure, 3 '→ 5'	Probe	*n*	Fluorescent melting		PCR-RT			
					Detection at 55°C		Detection at 64°C	
			*T*_m_ ,°C	SBR	relative background fluorescence, *I*_0MBi_/*I*_0MB1_	relative enhancement of fluorescence, *I*_fMBi_/*I*_0MBi_	relative background fluorescence, *I*_0MBi_/*I*_0MB1_	relative enhancement of fluorescence, *I*_fMBi_/*I*_0MBi_
	**MB1**	1	65.3	10.4	1.00	2.69	1.00	1.35
	**MB2**	2	70.9	11.3	0.74	2.97	0.89	1.63
	**MB3**	1	65.6	21.6	1.31	3.17	1.17	1.72
	**MB4**	2	71.8	7.7	1.31	1.49	0.75	1.21
	**MB5**	1	66.9	8.5	0.94	2.32	0.92	1.30
	**MB6**	2	68.5	9.1	0.88	2.07	0.52	1.40
	**MB7**	1	66.6	3.0	2.91	1.26	–	–
	**MB8**	2	73.3	4.6	0.95	1.60	0.57	1.26

Melting points of the probes (*T*_m_) were determined using heating in a thermal cycler (20°C → 95°C, see the Experimental section) defined as the value at the maximum point of the first derivative. Signal-to-Background Ratio (SBR) values are calculated as described in the Experimental section. *I*_0MB1_, initial (background) fluorescence intensity of probe **MB1**; *I*_0MBi_, initial (background) fluorescence intensity of probes **MB1**–**MB8**; *I*_fMBi_, final (plateau) fluorescence intensity of the probes **MB1**–**MB8**; values calculated based on RT-PCR experiments (for conditions see the Experimental section). All values are given as averages of parameters obtained in triplicate for each experiment. Dash means no data available. Q_*n*_ is the quencher (one or two, see value of *n*), L, linker; B, the branching fragment based on 3,5-diaminobenzoic acid.

When the hairpin probes are heated, the stem “melts” in the solution, leading to a flare-up of fluorescence. From the maximum of the first derivative of the function of the dependence of fluorescence intensity on temperature, one can determine the melting point of the probe (Table 1). It can be seen that the structure of the modification of the 5'-end region (linker and the number of 5-FAM residues) has almost no effect on the melting temperature of the probe. On the contrary, introduction of an additional quencher residue BHQ1 (Q) into the 3'-end region increases the melting point of the hairpin by 4–5°C, which is consistent with previously published data [14]. It is difficult to infer any regularities in fluorescence enhancement of the probes during melting; it can only be noted that probes with short linkers give a greater increase of emission intensity (Table 1).

Under RT-PCR conditions, with the accumulation of the PCR product, an increasing portion of the probe forms a duplex with it, and upon heating, the probe-target complex melts. Therefore, the change in fluorescence upon melting of the duplex of the probe with the target was also studied (see Supplementary Information). When melting both the hairpin and the duplex with the target, probe **MB2** with two quenchers showed the best melting cooperativity.

**Fig. 3.**
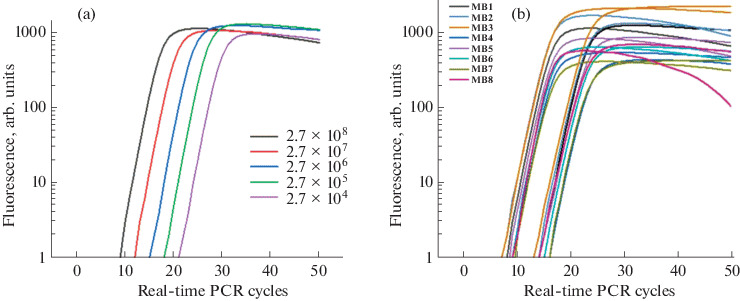
Fluorescence enhancement profiles in quantitative RT-PCR; fluorescence detection at 55°C. (a) Detection of different numbers of target molecules using probe **MB1**; (b) comparison of probes **MB1**–**MB8** in RT-PCR with 2.7 × 10^7^ (left) and 2.7 × 10^8^ target molecules (right).

Then the probes were compared under RT-PCR conditions, and fluorescence detection in each cycle was carried out at two temperatures (55 and 64°C). Background fluorescence values relative to control probe **MB1** ans the relative increase in fluorescence at the endpoint of the PCR in comparison with the initial fluorescence of the probe were obtained (Table 1). It can be seen that the additional quencher residue in all cases leads to some decrease in the initial (background) fluorescence (Table 1), and reduces the final flare-up of emission in the case of probes **MB4** and **MB6** (Fig. 3b).

To test the applicability of probes for quantitative detection of genetic material, a 10-fold dilution series of the target was used. Probe **MB1** allows target detection in the range of 50 zeptomol to 0.5 femtomol (Fig. 3a). The best sensitivity was demonstrated by probes **MB2** (doubled quencher and single 5-FAM residue on a short linker) and **MB3** (single quencher and FAM-bifluorophore on a short linker), they flared up about one cycle earlier than the standard probe **MB1**. The rest of the probes were inferior in sensitivity to **MB1** (Fig. 3b).

## EXPERIMENTAL

All solvents and reagents were used without further purification. 3-Azidopropylamine [21], as well as compounds (**II**) and (**IV**) [20, 22] were synthesized as previously described. ^1^H- and ^13^C-NMR spectra were obtained on a DRX-500 NMR spectrometer (500 MHz; Bruker, Germany) with assignment of signals by the peaks of residual protons in DMSO-*d*_6_ (2.50 ppm for ^1^H and 39.52 ppm for ^13^C). J-Coupling constants are given in hertz (Hz) for the corresponding multiplets. Thin layer chromatography was performed on TLC Silica gel 60 F_254_ aluminum plates (Merck, Germany).

**3-Azidopropyl-3,5-bis (3',6'-dihydroxy-3-oxo-3*H*-spiro(isobenzo-furan-1,9'-xanthene)-5-ylcarboxyamido)benzoate (V).** To acid solution (**II**) (2.00 g, 5.8 mmol) in DMF (15 mL), 3-azidopropylamine (700 mg, 6.9 mmol), EDC HCl (1.67 g, 8.7 mmol), HOBt (1.18 g, 8.7 mmol), Et_3_N (2.42 mL, 17.4 mmol) were added, and the reaction mixture was stirred for 12 h at room temperature under argon. Then the reaction mixture was diluted with AcOEt (50 mL), washed with water (2 × 30 mL), 10% citric acid solution (3 × 30 mL), 5% NaHCO_3_ solution (3 × 30 mL), saturated NaCl solution (30 mL), dried over Na_2_SO_4_, evaporated, and the residue was recrystallized from methylene chloride. 3,5-Di(trifluoroacetylamino)benzoic acid 3-azidopropylamide (2.00 g, 81%) was obtained as a white powder. *R*_f_ 0.6 (methanol-dichloromethane 5 : 95 (v/v)). Intermediate (**II**) ^1^H NMR (DMSO-*d*_6_), δ, ppm: 11.54 (s, 2H), 8.62 (t, *J* 5.6, 1H), 8.24 (t, *J* 2.0, 1H), 7.94 (d, *J* 2.0, 2H), 3.41 (t, *J* 6.8, 2H), 1.78 (quintet, *J* 6.8, 2H).

To a solution of bistrifluoroacetamide (**II**) (500 mg, 1.2 mmol) in methanol (15 mL) K_2_CO_3_ (842 mg, 6.1 mmol) and water (4 mL) were added, and the reaction mixture was stirred for 1.5 h while refluxing in an argon atmosphere. The reaction mixture was evaporated to dryness, evaporated with acetonitrile (4 × 20 mL), dissolved in AcOEt (30 mL), dried over Na_2_SO_4_, and evaporated. Compound (**III**) was obtained as a yellowish oil, and further used without additional purification and characterization. *R*_f_ 0.32 (methanol-dichloromethane 1 : 9 (v/v)).

Compound (**III**) was dissolved in DMF (20 mL), DMAP (428 mg, 3.5 mmol), Et_3_N (488 μL, 3.5 mmol), pentafluorophenyl ether of pivalate-protected carboxyfluorescein (**IV**) (2.08 g, 2.9 mmol) were added, and the reaction mixture was stirred for 7 days at 50°C in an argon atmosphere. Then the reaction mixture was evaporated to dryness, diluted with AcOEt (50 mL), washed with water (2 × 30 mL), 10% citric acid (2 × 30 mL), 5% NaHCO_3_ solution (3 × 30 mL), and saturated NaCl solution (30 mL). The organic phase was dried over Na_2_SO_4_, evaporated, and the residue was chromatographed on silica gel in the dichloromethane/acetone system (100 : 0 → 75 : 25 (v/v)). The target fractions were combined, evaporated, the residue was dissolved in acetone (15 mL), 25% aqueous ammonia solution (2 mL) was added, and the mixture was stirred for 1 h at room temperature. Excess ammonia and acetone were evaporated, the solution was diluted to 10 mL with water and adjusted with a 10%-HCl solution to pH 2; the precipitate was filtered and washed with water. It was recrystallized from a mixture of methanol and acetonitrile (1 : 9 (v/v)). Compound (**V**) was obtained as a yellowish powder (645 mg, 58%). *R*_f_ 0.7 (methanol–ethyl acetate 1 : 3 (v/v). ^1^H-NMR (DMSO-*d*_6_), δ, ppm: 10.82 (s, 2H), 8.69-8.65 (m, 2H), 8.62-8.57 (m, 2H), 8.40 (dd, *J* 8.1, 1.7, 2H), 8.02 (d, *J* 1.9, 2H), 7.47 (d, *J* 8.1, 2H), 6.75 (d, *J* 2.3, 2H), 6.64 (d, *J* 8.7, 4H), 6.62–6.58 (m, 4H), 3.44 (t, *J* 6.8, 2H), 3.36 (qt, *J* 6.4, 2H), 1.82 (quintet, *J* 6.8, 2H). ^13^C NMR (DMSO-*d*_6_), δC, ppm: 168.15, 166.62, 164.12, 159.91, 154.93, 151.97, 139.14, 136.42, 135.96, 135.23, 129.22, 126.61, 124.55, 124.11, 115.56, 115.46, 112.90, 109.14, 102.40, 48.62, 36.83, 28.47.

**Quantitative RT-PCR** was carried out on a DTprime detecting amplifier (DNA technology, Russia) using a pair of primers Fat65R–Fat65F and plasmid pTZ-Fat containing a 290-bp fragment of the gene encoding translation elongation factor 1α of *Fusarium avenaceum* (Fig. 2d).

The reaction mixture (35 μL) contained 83.75 mM Tris-HCl, 20.75 mM ammonium sulfate, 3.125 mM magnesium chloride, 0.003% Tween-20, 0.003% NP-40, 6.25% glycerol, 0.17 mM of each dNTP, 0.36 μM primers, 0.2 μM of each probe from the set **MB1**–**MB8**, 2.5 IU Taq polymerase and DNA template (plasmid pTZ-Fat, 2.7 × 10^4^–2.7 × 10^8^ copies), pH 8.8. The experiments were repeated three times and analyzed by the geometric method (C_q_) using the Real-time PCR 7.9 software (DNA Technology, Russia). The background fluorescence of each sample well was taken into account. For quantitative detection of different matrix concentrations with an **MB1** probe, a direct dependence of the number of cycles on the matrix content in the sample was observed, with *R*^2^ ≥ 0.99. For quantitative PCR, a matrix solution with a predetermined concentration (measured spectrophotometrically at a wavelength of 260 nm) was used. Aliquots were taken from this solution and samples with a known number of target molecules were obtained by subsequent dilution. RT-PCR program: 80°C for 60 s; 94°С for 90 s (1 cycle); then 94°C for 30 s, 64°C for 15 s (5 cycles) and 94°C for 10 s, 64°C for 15 s (45 cycles) (fluorescence was recorded at 55 or 64°C using a detector in the FAM-channel).

**Melting experiments on probes MB1**–**MB8** were carried out in three repetitions on the same device (FAM detection channel) with each probe from the row **MB1**–**MB8** melted separately in a PCR buffer without dNTPs, primers, Taq polymerase and plasmid template. The concentration of each probe was 0.2 μM. The temperature was increased from 20 to 95°C in 0.5°C steps for 15 s. For each step, the fluorescence level was measured. MB_i_/target duplexes were preliminarily annealed with a twofold excess of a short 26-mer complementary sequence and melted under the same conditions. Then, a graph of the dependence of the fluorescence intensity on temperature in the range of 20–95°C was constructed. Melting points were calculated as the maxima of the first derivative using the OriginPro 8 software. The signal-to-background ratio (SBR) for probes **MB1–MB8** was calculated by the formula:

$${\text{SBR}} = {{\left( {{{I}_{{{\text{fMBi}}}}}-{{I}_{0}}} \right)} \mathord{\left/ {\vphantom {{\left( {{{I}_{{{\text{fMBi}}}}}-{{I}_{0}}} \right)} {\left( {{{I}_{{0{\text{MBi}}}}}-{{I}_{0}}} \right)}}} \right. \kern-0em} {\left( {{{I}_{{0{\text{MBi}}}}}-{{I}_{0}}} \right)}},$$

where *I*_0_ is the fluorescent noise signal of a PCR sample containing all components, but without a probe; MB_i_, a test probe from the set **MB1–MB8**; *I*_0MBi_, fluorescence MB_i_ in the absence of a matrix; *I*_fMBi_, the fluorescence signal of prehybridized MB_i_ with a short complementary 26-mer sequence 5'-CGGGGTAATGAATGCGTTTCGAATGA-3'. Measurements of the values of *I*_0_, *I*_0MBi_, *I*_fMBi_ were carried out at 20°C in a DTprime detecting amplifier (DNA Technology, Russia) with 0.2 μM MB_i_ and a twofold excess of the complementary oligonucleotide.

## CONCLUSIONS

In this work, we investigated the efficiency of a FAM-bifluorophore based on 3,5-diaminobenzoic acid on various linkers compared to a single fluorescein label as part of fluorogenic molecular beacon oligonucleotide probes for RT-PCR. It has been shown that for such hairpin RT-PCR probes, it is preferable to attach fluorescein (both a single label and a bifluorophore) through a short linker using amidophosphite reagents. The highest sensitivity of the probe in RT-PCR is achieved with the introduction of nonstandard modifications a doubled quencher or a doubled dye, but not both modifications simultaneously.

## Supplementary Information

11171_2021_8334_MOESM1_ESM.pdf
