# Supplementary material for: Molecular Beacon DNA Probes with Fluorescein Bifluorophore
Source: Russ J Bioorg Chem. 2021 Jun 11;47(3):734–40. doi: 10.1134/S1068162021030055 (PMC8193957; doi:10.1134/S1068162021030055)
Supplement: Supplementary file 1 — 11171_2021_8334_MOESM1_ESM.pdf [file 11171_2021_8334_MOESM1_ESM.pdf]

SUPPLEMENTARY MATERIALS

Molecular Beacon DNA Probes with Fluorescein Bifluorophore

V. A. Brylev*, #, I. L. Lysenko, E. A. Kokin*, Y. V. Martynenko-Makaev**, D. Y. Ryazantsev*,**

V. V. Shmanai, and V. A. Korshun*, ***, ******

#Phone: +7(499)724-67-15; e-mail: v.brylev@yandex.ru

**Shemyakin-Ovchinnikov Institute of Bioorganic Chemistry, ul. Miklukho-Maklaya 16/10, Moscow, 117997 Russia*

***Institute of Physical Organic Chemistry of NAS Belarus, ul. Surganova 13, Minsk, 220072 Belarus*

****Department of Biology and Biotechnology, National Research University Higher School of Economics,*

ul. Vavilova 7, Moscow, 117312 Russia

*****Gause Institute of New Antibiotics, ul. Bolshaya Pirogovskaya 11, Moscow, 119021 Russia*

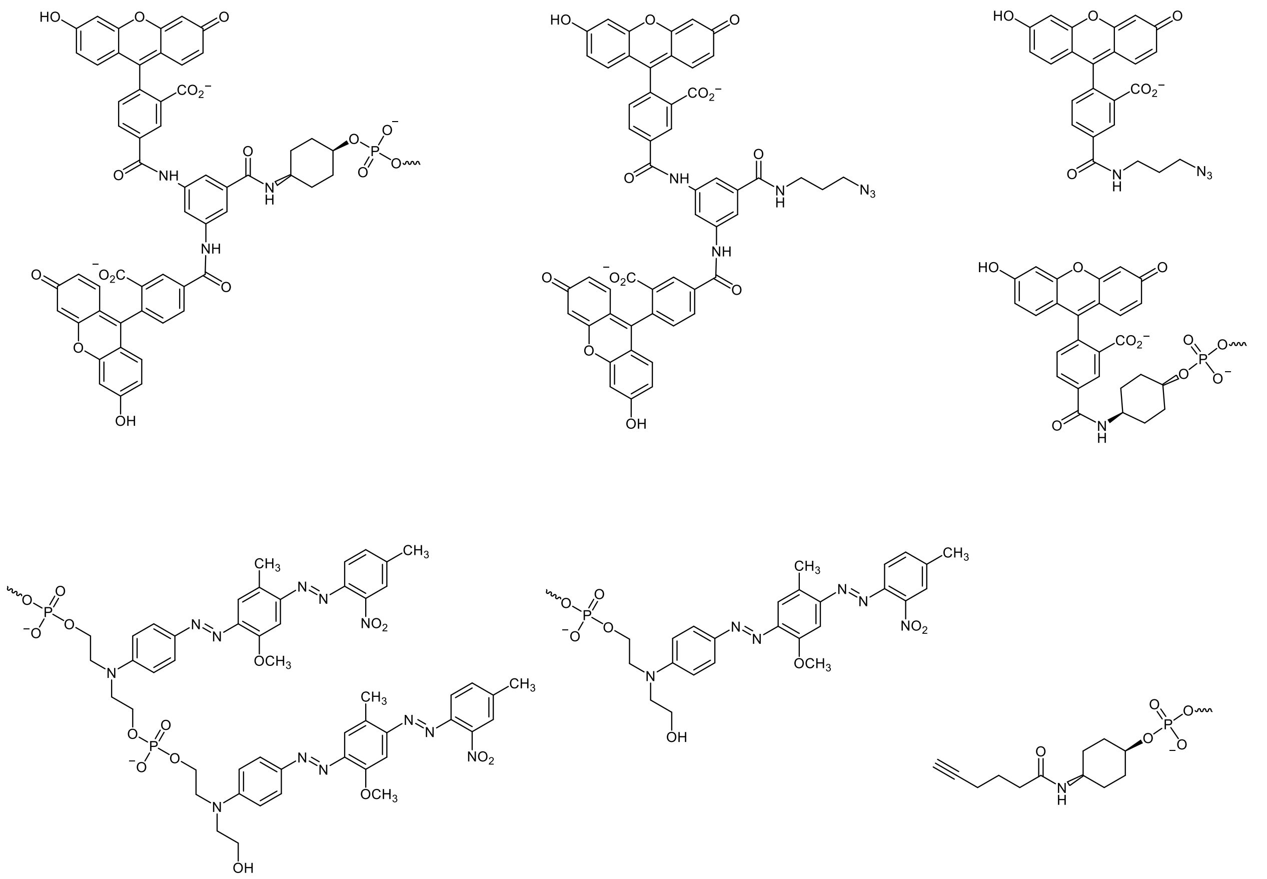

Fig. S1. 5'-Modifications used in this study.

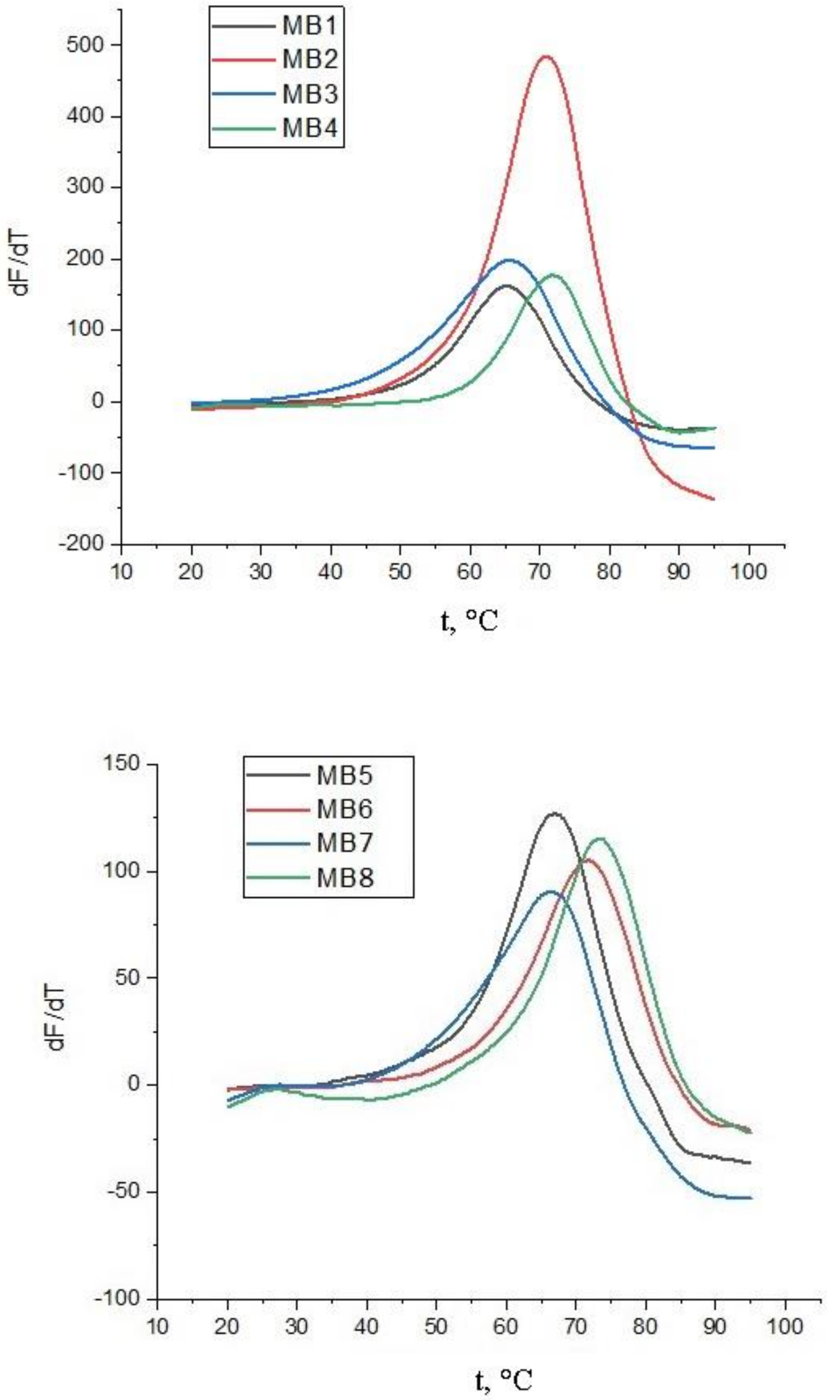

Fig. S2. Melting curves of hairpin probes MB1 – MB8 (heating from 20 to 95 ºC).

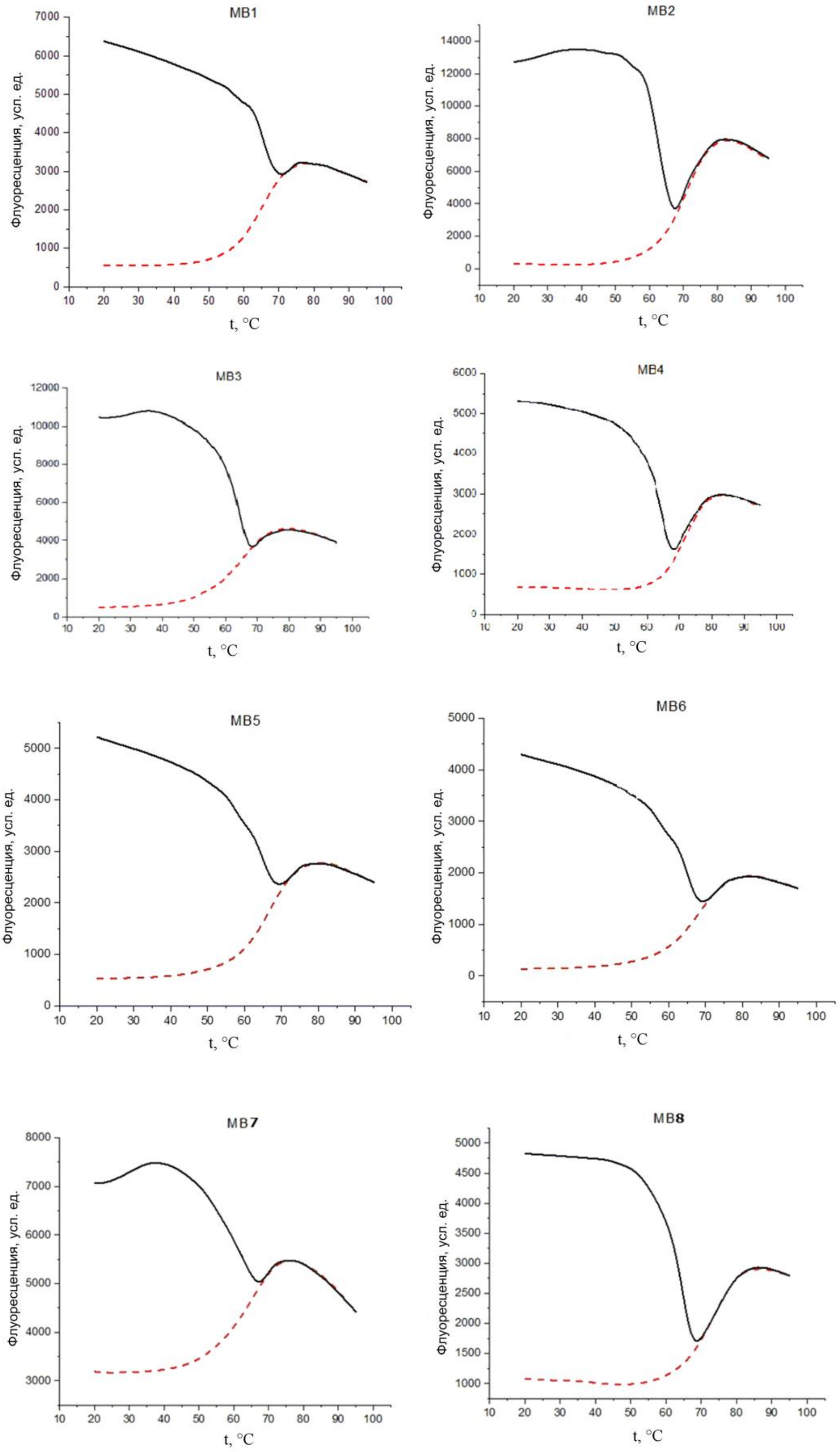

Fig. S3. Melting curves of probes MB1 – MB8 (solid line — duplex with a 26-mer DNA template, dashed line — hairpin).
